# SIRT1 activity orchestrates ECM expression during hESC‐chondrogenic differentiation

**DOI:** 10.1096/fj.202200169R

**Published:** 2022-04-13

**Authors:** Christopher A. Smith, Paul A. Humphreys, Nicola Bates, Mark A. Naven, Stuart A. Cain, Mona Dvir‐Ginzberg, Susan J. Kimber

**Affiliations:** ^1^ Division of Cell Matrix Biology and Regenerative Medicine School of Biological Sciences University of Manchester Manchester UK; ^2^ Laboratory of Cartilage Biology Faculty of Dental Medicine Hebrew University of Jerusalem Jerusalem Israel

**Keywords:** chondrogenesis, embryonic stem cells, human pluripotent stem cells, matrix, SIRT1

## Abstract

Epigenetic modification is a key driver of differentiation, and the deacetylase Sirtuin1 (SIRT1) is an established regulator of cell function, ageing, and articular cartilage homeostasis. Here we investigate the role of SIRT1 during development of chondrocytes by using human embryonic stem cells (hESCs). HESC‐chondroprogenitors were treated with SIRT1 activator; SRT1720, or inhibitor; EX527, during differentiation. Activation of SIRT1 early in 3D‐pellet culture led to significant increases in the expression of ECM genes for type‐II collagen (*COL2A1*) and aggrecan (*ACAN*), and chondrogenic transcription factors *SOX5* and *ARID5B*, with SOX5 ChIP analysis demonstrating enrichment on the chondrocyte specific –10 (A1) enhancer of ACAN. Unexpectedly, when SIRT1 was activated, while ACAN was enhanced, glycosaminoglycans (GAGs) were reduced, paralleled by down regulation of gene expression for N‐acetylgalactosaminyltransferase type 1 (*GALNT1)* responsible for GAG chain initiation/elongation. A positive correlation between *ARID5B* and *COL2A1* was observed, and co‐IP assays indicated association of ARID5B with SIRT1, further suggesting that *COL2A1* expression is promoted by an ARID5B‐SIRT1 interaction. In conclusion, SIRT1 activation positively impacts on the expression of the main ECM proteins, while altering ECM composition and suppressing GAG content during human cartilage development. These results suggest that SIRT1 activity has a differential effect on GAGs and proteins in developing hESC‐chondrocytes and could only be beneficial to cartilage development and matrix protein synthesis if balanced by addition of positive GAG mediators.

**Abbreviations**
ACAN: aggrecan gene
ADAMTS4: A disintegrin and metalloproteinase and thrombospondin motifs 4
ADAMTS5: A disintegrin and metalloproteinase and thrombospondin motifs 5
ARID5B: AT‐rich interaction domain 5B
AROS: active regulator of SIRT1
BMP2: bone morphogenetic protein
CB: chondro‐basal
cDNA: complementary deoxyribonucleic acid
ChIP: chromatin immunoprecipitation
COL1: type I collagen
COL2: type II collagen
COLX: type X collagen
DDP: defined differentiation protocol
DMNT1: DNA methyltransferase 1
DMSO: dimethyl sulfoxide
DNA: deoxyribonucleic acid
DOX: doxycycline
kb: kilobase
EC60: 60% of maximal effective concentration
ECM: extracellular matrix
EDTA: ethylenediaminetetraacetic acid
EF1a: elongation factor 1
FBS: fetal bovine serum
FGF2: fibroblast growth factor 2
GAG: glycosaminoglycan
GALNT1: N‐acetylgalactosaminyltransferase 1
GAPDH: glyceraldehyde 3‐phosphate dehydrogenase
GDF5: growth differentiation factor 5
GFP: green fluorescent protein
HA: hyaluronic acid
HABP: hyaluronan binding protein
HAPLN1: hyaluronan and proteoglycan link protein 1
HAS2: hyaluronan synthase 2
HDAC2: histone deacetylase 2
HDAC4: histone deacetylase 4
hESC: human embryonic stem cell
hPSC: human pluripotent stem cell
iMSC: Induced mesenchymal stromal cell
iPSC: induced pluripotent stem cell
ITS: Insulin, transferrin, selenium
KDM2B: lysine demethylase 4B
KDM4B: lysine demethylase 4B
KO: knock out
MCT: microcentrifuge tube
MSC: mesenchymal stromal cell
NAD: nicotinamide adenine dinucleotide
PBS: phosphate buffered saline
PBS‐T: phosphate buffered saline Tween 20
pH: potential of hydrogen
PHF2: PHD finger protein 2
PPFA: paraffin‐paraformaldehyde
QRT‐PCR: quantitative real time polymerase chain reaction
RCF: relative centrifugal force
RIPA: radioimmunoprecipitation assay
RNA: ribonucleic acid
RT‐PCR: reverse transcription polymerase chain reaction
RUNX2: runt related transcription factor 2
SETD7: SET domain containing 7
shRNA: short hairpin ribonucleic acid
SIRT1/2/3/6/7: Sirtuin 1/2/3/6/7
SOX5/6/9: SRY‐box transcription factor 5/6/9
TCP: tissue culture plastic
TET: tetracycline
TGFβ3: transforming growth factor β3
XYLT1: xylosyl‐transferase 1
XYLT2: xylosyl‐transferase 2

## 1 INTRODUCTION

Human embryonic stem cells (hESCs) and induced pluripotent stem cells (iPSCs), together termed human pluripotent stem cells (hPSCs), have great potential for understanding human development including that of skeletal tissues, because of their self‐renewal properties and ability to differentiate into many different tissue lineages.^1^, ^2^ We have previously reported the differentiation of chondroprogenitors and chondrocytes from hESCs in a 3 stage serum‐free protocol.^3^ We utilized a series of growth factors based on developmental principles,^4^, ^5^ starting with transient exposure to the Wnt agonist CHIR99021 and Activin A^6^ and then using BMP2 with later addition of GDF5,^4^, ^5^ important in joint cartilage development. The cells generated express the key chondrogenic transcription factors SOX 9, 5 and 6 as well as the archetypal matrix component type‐II collagen. Type‐II collagen is the main fibrillar collagen found in cartilage and is responsible for its tensile strength. In addition to type‐II collagen there are multiple supportive collagens (e.g. type IV, IX and XI) as well as many proteins decorated with GAGs. The most important GAG‐associated protein in cartilage is aggrecan, responsible for its water retention and load‐bearing properties. Additionally, in keeping with a hyaline‐like differentiation and the generation of articular cartilage, type‐X collagen (*COLX*) is undetectable, indicating that hypertrophic chondrocytes of the growth plate are not being generated. While promising, the expression of the hyaline cartilage proteoglycan aggrecan in the matrix remains low suggesting incomplete matrix synthesis and immaturity, so it is important to remedy this to produce a valid developmental model.

Epigenetic effectors can regulate gene expression by modifying histones, as well as various transcription factors. Therefore, modulation of these epigenetic effectors may influence gene expression in chondroprogenitors. The NAD dependent deacetylase, SIRT1 is an epigenetic effector capable of deacetylating histones, and non‐histone proteins.^7^, ^8^ Importantly, the major transcription factor driving cartilage formation, SOX9^9^, ^10^ is a target of SIRT1,^11^, ^12^ which deacetylates it, promoting chondrogenic activity.^13^

While much is known about the role of SIRT1 in cartilage homeostasis and disease, much less is known about its role in development. SIRT1 expression is required for the maintenance of hyaline cartilage, and chondrocyte differentiation^12^, ^13^ and has been identified as a pro‐survival and ‐metabolic factor, maintaining the homeostasis of the adult chondrocyte in its niche.^14^ Importantly, *Sirt1* ^−/−^ knock out mice display skeletal and cartilage matrix deficiencies,^15^, ^16^, ^17^ indicating a positive role of SIRT1 in articular cartilage homeostasis. However, although cartilage is formed in this KO model, an involvement of SIRT1 in the initial expression of cartilage ECM components during development is not clear. SIRT1 may play a role in homeostasis by repressing catabolic gene expression^14^ while promoting anabolic pathways towards synthesis of collagen type‐II and aggrecan. However, there is a lack of knowledge of the timing and role of SIRT1 in human cartilage development. Using RNAseq analysis of hESC‐chondroprogenitors, we identified the presence of SIRT1 alongside significant increases in supportive chondrogenic transcription factors, SOX5 and AT‐Rich Interaction Domain 5B (ARID5B), suggesting their importance.^18^ SOX5 and ARID5B are both able to form complexes with SOX9 to facilitate chondrocyte maturity. Specifically, SOX9 participates with SOX5 in the SOX trio^19^, ^20^, ^21^ to direct aggrecan expression, while ARID5B binds the epigenetic factor PHD Finger protein 2 (PHF2) to direct aggrecan and type‐II collagen expression.^22^ Here we assess the role of SIRT1 in regulating aggrecan and collagen type‐II expression during hESC chondrogenesis. Specifically, by modulating SIRT1 activity during the chondrogenesis protocol we start to decipher the pathway by which it regulates these key chondrogenic genes and its role in human cartilage development.

## 2 EXPERIMENTAL PROCEDURES

### 2.1 hESC culture

The human embryonic stem cell lines Man‐7 and Man‐13^23^, ^24^ were cultured under feeder‐free conditions in mTeSR1 (StemCell Technologies, France), on 6 well tissue culture plastic (TCP) plates (Corning, UK) pre‐coated with 5 µg/ml Vitronectin (N‐Terminal fragment Life Technologies, USA). Cells were passaged at 80% confluency using 0.5 mM EDTA and plated with 10 nM ROCK inhibitor Y27632 (Tocris, UK) exposure for a maximum of 24 h after a split. For experimental use, hESCs were passaged at 100,000 cells /cm^2^ into a 6 well plate pre‐coated with fibronectin (Millipore, USA). Differentiation protocols were started when cells were 70–80% confluent.

### 2.2 Directed differentiation protocol

**FIGURE 1:**
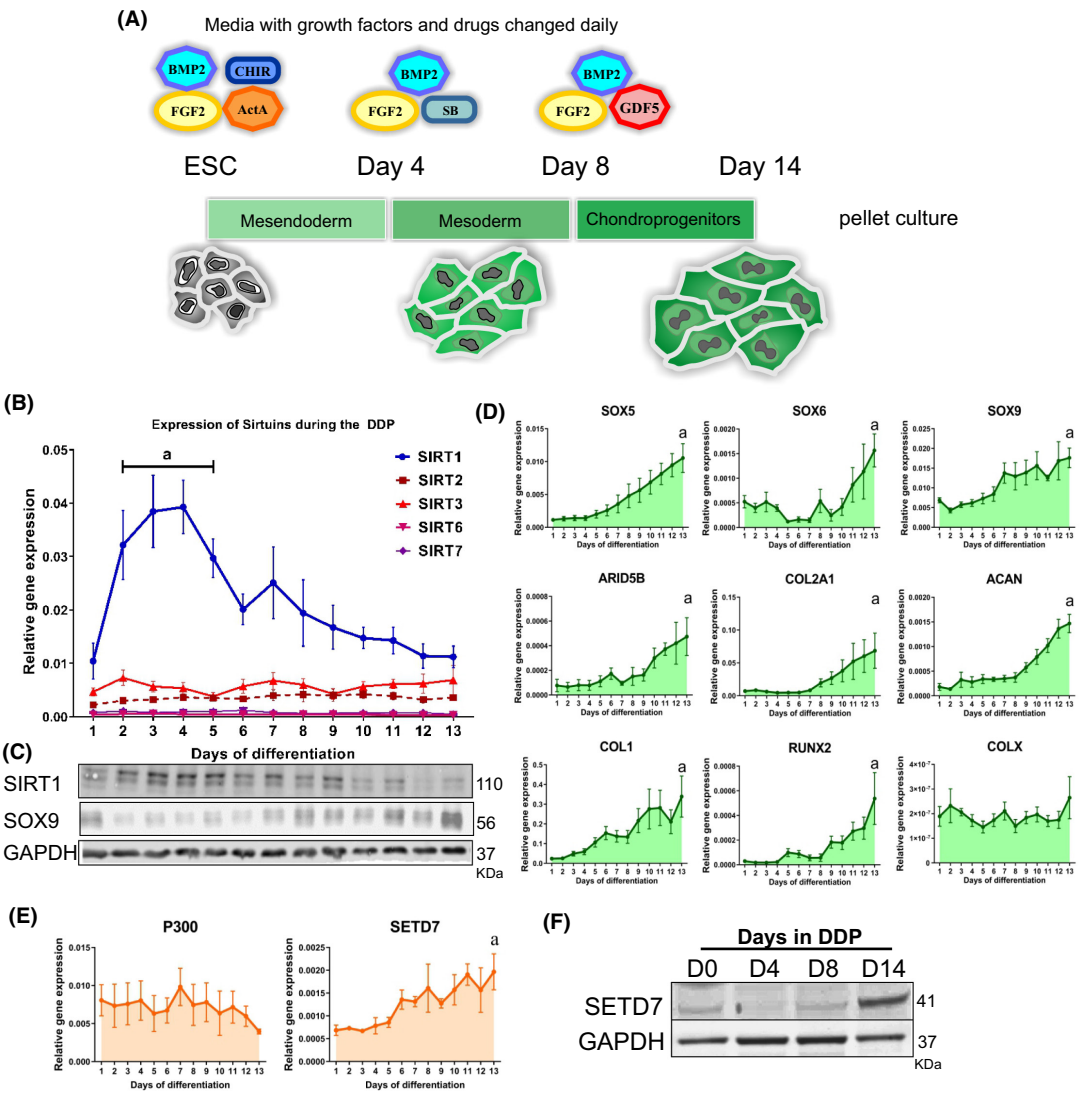
SIRT1 expression changes during the chondrogenic differentiation of hPSCs to chondroprogenitors. (A) Schematic diagram of 2D phase of DDP differentiation protocol (B) QRT‐PCR gene expression analysis of Sirtuins 1,2,3,6 and 7 in samples taken daily (D1–13) over the 13 days of the DDP chondrogenic differentiation process (*N* = 4 biological repeats). (C) Western blot analysis of protein expression for SIRT1, SOX9, and the housekeeping control gene GAPDH in samples taken daily from the chondrogenic DDP protocol. Samples correspond to days displayed in panel (B) above. (D) QRT‐PCR gene expression analysis of transcripts associated with formation of permanent cartilage (*SOX5*, *SOX6*, *SOX9*, *ARID5B*, *COL2A1* and *ACAN*) and other fibrocartilage or hypertrophic associated phenotypes (*COL1*, *RUNX2*, *COLX*) in samples taken daily from the DDP chondrogenic protocol (*N* = 4 biological repeats). (E) QRT‐PCR gene expression and western blot (F) for protein expression of epigenetic factors associated with chondrogenic regulatory mechanisms. Data displayed as relative gene expression to housekeeping gene *GAPDH* and shown in box plot. *a* indicates a significant difference to day 0 (*p* ≤ .05)

Chondroprogenitors were produced by differentiating hESCs through a defined differentiation protocol (DDP) (Figure 1A). For chondrogenic differentiation hESCs were differentiated in a basal medium (DMEM:F12, 2 mM L‐glutamine (Life Technologies), 1% (vol/vol) insulin, transferrin, selenium (ITS) (Sigma, UK), 1% (vol/vol) non‐essential amino acids (Thermo), 2% (vol/vol) B27 (Gibco), 90 μM β‐mercaptoethanol) (Gibco) supplemented with appropriate sequential addition of growth factors as previously described^3^, ^5^ with the following stages; Stage 1 (day 1–3):CHIR (2 µM, R&D systems, UK), Activin‐A (reducing from 50 to 10 ng/ml, Peprotech, UK) and BMP2 (concentration set at EC60 for consistent activity; R&D systems), followed by Stage 2 (day 4–8): BMP2 (See above), SB431542 (1 mM) and GDF5 (20 ng/ml) (all Peprotech) and finally Stage 3 (day 9–14): GDF5 (20 rising to 40 ng/ml at day 11), FGF2 (20 ng/ml) ml (both Peprotech), with BMP present at half stage 1 concentration until day 11. At days 4 and 8, cells were split 1 to 4 using EDTA and TrypLE respectively. To understand the expression of SIRT1 at each day during the 14 day protocol, initially samples were taken daily. After the expression profile of SIRT1 was identified, subsequent samples were collected for RNA and protein at day 4, 8 and 14.^4^

Cells cultured in the above medium were supplemented with either DMSO vehicle control (1 µl/ml), SIRT1 enzyme activator SRT1720 (Selleckchem, UK)^25^ (concentration specified in figure legend), or SIRT1 inhibitor EX527 (Sigma)^13^, ^26^ (concentration specified in figure legend). During the 2D stage of the protocol, medium is changed daily. As such, drugs were added daily in media changes at either days 2–5, days 9–14, or during pellet culture (below) as indicated in Results.

### 2.3 Pellet formation

**FIGURE 2:**
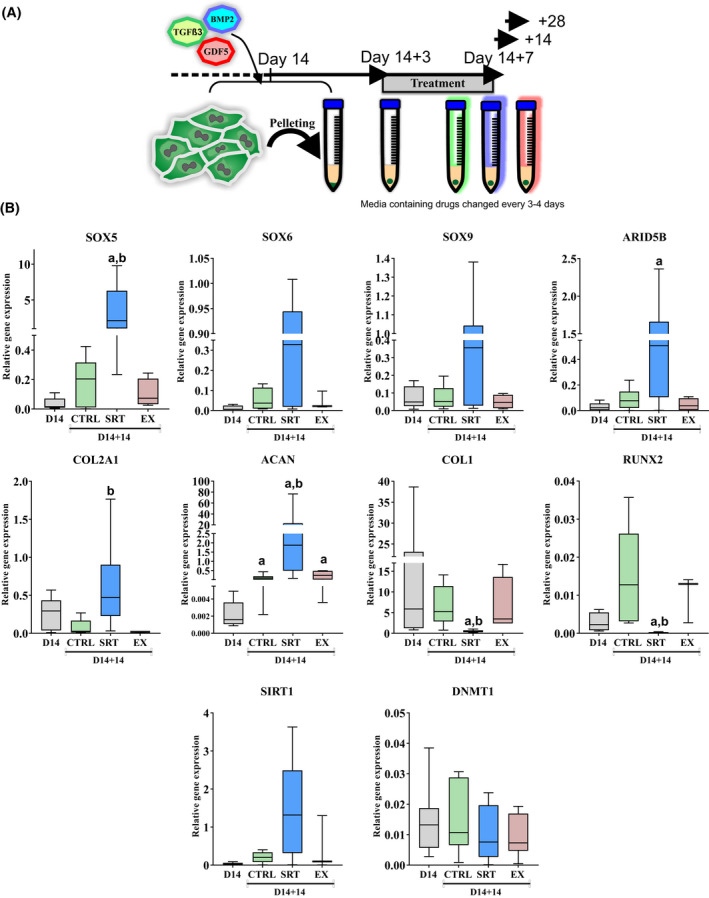
SIRT1 activation in hPSC derived pre‐chondrocyte pellets modulates chondrogenic ECM expression. (A) Schematic of pellet formation and 3D culture phase of DDP differentiation protocol. (B) QRT‐PCR gene expression analysis of chondrogenic and non‐chondrogenic associated genes in 2D day 14 pre‐pellet cells, and pellets at day 14 + 14, treated with DMSO (control) or SIRT1 activity modulators from day 14 + 3 to 14 + 14 (*N* = 7 biological repeats). Data displayed as gene expression relative to housekeeping gene *GAPDH* and shown in box plot. *a* indicates significant difference to Day 14 pre‐pellet sample (*p* ≤ .05), *b* indicates significant difference to day14 + 14 DMSO vehicle (control) (*p* ≤ .05)

At day 14 cells were separated from coated tissue culture plates using TrypLE (Life Technologies) and counted using a nucleocounter (Chemometech, Denmark). Cells were resuspended at 5 × 10^5^ cells/ml in the same medium used at day 14. For each pellet a 1 ml aliquot of 5 × 10^5^ cells was pipetted into a 15 ml centrifuge tube and centrifuged at 300 xRCF for 3 min to sediment cells; caps were left loose, and pellets were incubated at 37°C 5% CO_2_ for 3 days. Pellets were then transferred to chondro‐basal medium (CB) containing Dulbecco's Modified Eagle Medium (DMEM) (Life Technologies), L‐glutamine (2 mM), L‐Proline (40 µg/ml) (Sigma), 1x ITS (Sigma), ascorbate‐2‐phosphate (50 µg/ml) (Sigma), and dexamethasone (100 nM) (Sigma) supplemented with TGFβ3 (10 ng/ml) (Peprotech), GDF5 (20 ng/ml) (Peprotech) and BMP2 (half concentration of stage 1) (R&D systems). Pellets were separated into 4 groups and incubated with the above medium containing DMSO vehicle control (1 µl/ml), 5 µM SRT1720 (concentration based on results described in Supplementary Figure 2A), and 5 µM EX527 as indicated in Results. Drugs were added with each change of medium, every 3 to 4 days until either 14‐ or 28‐days post pelleting (See Figure 2A).

### 2.4 TC28a2 cell culture

Immortalized chondrocytes, TC28a2 cells,^27^ were cultured in DMEM containing 10% FBS, and L‐glutamine (2 mM). Cells were cultured on TCP in 2D. For 3D culture, cells were trypsinized, re‐suspended in TC medium at 1 × 10^6^ cells/ml, and centrifuged at 300 xRCF for 5 min. Pellets were incubated for 3 days, and then the medium was changed to CB medium as described in previous section.

### 2.5 Gene transcription analysis

Cells were transferred to RLT buffer directly and RNA extracted by use of a RNeasy QIAgen kit according to manufacturers’ instructions. For 3D cultures, pellets were first dissociated by grinding with Molecular Grinding Resin™ (Sigma) with a pestle in a 1.5 ml microcentrifuge tube (MCT). The ground pellets were then mixed with RLT buffer and RNA extracted. Extracted RNA was DNase treated and reverse transcription polymerase chain reaction (RT‐PCR) used to produce cDNA with ABI‐RT kit (Life technologies). Quantitative real‐time polymerase chain reaction (QRT‐PCR) was conducted using PowerUp™ SYBR™ green (Life technologies) and primers for chondrogenic and non‐chondrogenic genes (see Table S1). Samples were initially heated to 95°C for 10 min and then run for 40 cycles of 95°C for 30s, 60°C for 30s, 72°C for 35s with a final elongation step of 72°C for 10 min. Data was calculated and displayed as relative expression to housekeeping gene *GAPDH*.

### 2.6 Western blotting and immunoprecipitation

Cell samples were lysed in RIPA buffer (Sigma) with added 1x Roche complete protease inhibitors (Sigma) and quantified using Pierce® BCA (Thermo). Aliquots containing 30 µg of protein were boiled at 95°C for 10 min. Samples were loaded on 10% SDS PAGE gels (Invitrogen). Protein gels were first blocked in 5% Marvel milk™ solution in PBS‐T before probing with specific primary antibodies (see Table S2). Antibody binding was detected using LI‐COR IRDye secondary antibodies. Blots were imaged using the Odyssey CLx. Quantification of protein was achieved using ImageJ, and protein quantification was calculated relative to GAPDH internal control levels.

For immunoprecipitation procedures, approximately 15–20 pellets were fixed for 1hr in 4% paraformaldehyde, then washed twice before being ground (as above). Material was lysed using 300 µl RIPA buffer containing PMSF (1 mM) and protease inhibitor cocktail (Roche). Samples were centrifuged at 15,000 xRCF for 10 min at 4°C, and supernatant assessed for protein concentration. A 100 µg aliquot was diluted to 300 µl and incubated overnight with either antibody to SIRT1 (Millipore) or to P300 (Abcam) at 4°C under constant agitation. After preliminary incubation, 50 µl of washed magnetic beads (Pierce) were added to the solution and incubated at 4°C for a further 5 hours. Supernatant was removed, and the beads washed 3 times in PBS before being boiled at 95°C for 10 min with reducing buffer (Thermo, 39000). Resulting samples was analyzed using western blotting with detection using anti‐rabbit IgG light chain antibody (Abcam) and ClarityMax ECL or DAB (BIO‐RAD and Sigma, respectively).

### 2.7 Chromatin immunoprecipitation (ChIP)

ChIP was conducted using the Diagenode LowCell ChIP kit (Diagenode). Chondrogenic pellets were washed in PBS and then fixed in 1% PFA at room temperature for 30 min. After incubation, glycine was added to a final concentration of 0.125 M, the solution was mixed and further incubated at room temperature for 5 min. Samples were then washed twice in ice cold PBS containing inhibitor cocktail, PMSF (1 mM supplier) and protease inhibitors (supplied by kit supplier), sodium butyrate (5 mM). Pellets were then ground using pestle and Molecular Grinding Resin (as above) and resuspended in 60 µL buffer B supplied in the kit. Samples were sonicated using the BioRupter (Diagenode) at full power for 30 cycles (30 seconds on; 30 seconds off). Chromatin was extracted using SIRT1 and SOX5 antibodies (see Table S2), and DNA isolated as described in kit. Isolated genomic DNA was analyzed using QRT‐PCR using primers for *COL2A1* promoter and enhancer, and *ACAN*‐10 enhancer (see Table S1).

### 2.8 Lentiviral overexpression

Stably expressing TC28a2 and Man‐13 cells were generated using an established lentiviral method (SBI Systems Bioscience). cDNA sequences used for lentiviral overexpression were based on that of human *SIRT1* (Gene ID: 23411) and were subcloned into a modified Doxycycline (Dox) inducible viral expression vector based on the pCDH‐EF1‐T2A‐copGFP vector. His‐Flag‐SIRT1 (gift from Prof. Danny Reinberg, NYU, NY), SIRT1‐shRNA, and *ARID5B*‐shRNA expression was controlled by the inducible TRES3G promoter. Tetracycline (Tet)‐On 3G trans‐activator protein and tagGFP2 fluorescent protein were under the constitutive promoter Elongation factor 1 (EF1a). This system is responsive to Tet and derivative Dox. HEK293T cells were co‐transfected with psPax2, pMD2.G packaging as well as a target pCDH vector. Production of virus particles was induced by addition of 10 mM sodium butyrate (Millipore) for 4 hours, 24 h post‐transfection, and virus particles were harvested from the medium after 48 hours and filtered through a 0.2 µm filter prior to addition to target cells. TC28a2 or Man‐13 cells were then infected with virus using 5 µg/ml protamine sulphate (Sigma). Cells were passaged, followed by fluorescence‐activated cell sorting (FACSAria Fusion, Beckon‐Dickenson), where applicable.

### 2.9 Dimethyl‐methylene blue (DMMB) sulphated GAG assay

DMMB assay was undertaken using the Blyscan™ assay kit (Biolcolor) to ascertain the sulphated GAG‐content of pellets. Pellets were first digested in 200 µl papain digestion solution containing PBS and 0.5 mg/ml papain (Sigma) overnight at 65°C with intermittent agitation. Samples were centrifuged at 10,000 xRCF for 10 min and supernatant decanted for storage before use. Digested samples were stored at −20°C until assayed.

For assay, 50 µl of sample (medium or lysate) was diluted with 500 µl of Blyscan dye reagent and incubated for 30 min. Samples were centrifuged at 12 000 xRCF for 10 min and resulting pellet resuspended in 500 µl dissociation reagent. 100 µl of samples was measured for absorption at 650 nm. A Quant‐iT pico‐green dsDNA assay (Thermo) was used to quantify DNA, which was used to standardize readings.

### 2.10 Histological assessment (IHC, IP)

Chondrogenic pellets were harvested at Day 14 monolayer culture, and at +14 days or +28 days in pellet culture, rinsed in PBS and fixed overnight in 4% paraformaldehyde at 4°C. After this, pellets were stored in 70% ethanol before processing. Processed samples were embedded in PPFA wax, sectioned to 5 µM, and deposited on glass slides.

Wax was removed by Xylene, and sections were rehydrated through serial alcohol solutions (100%, 90%, 70%) and then dH_2_O. Sections were washed 3 times in PBS + 0.1%Tween 20, then subjected to antigen retrieval with citrate buffer (pH6.5) and heated to 95°C, or Pepsin enzyme (Thermo). Sections were first blocked using primary animal serum, then incubated overnight at 4°C with primary antibodies as described in results (see Table S2), with antibody detection by either biotinylated secondary followed by streptavidin peroxidase or fluorochrome tagged secondary antibodies as appropriate. No primary or inappropriate primary antibodies were used as control. A hyaluronic acid binding protein (HABP) antibody was used to visualize the polysaccharide hyaluronic acid.

### 2.11 Statistical analysis

All statistical analysis was run using Prism Graph‐pad. Gene and protein expression changes were analyzed using Mann–Whitney *U* test. ChIP was analyzed using a ratio paired t‐test. Correlation analysis was quantified using a Pearson's correlation coefficient. A *p*‐value of ≤.05 was considered as statistically significant.

## 3 RESULTS

### 3.1 SIRT1 expression is dynamic, and is elevated during early stages of chondrogenic differentiation

To understand the importance of SIRT1 in early chondrogenic development, we first sought to determine its expression during the generation of chondroprogenitors, using the 14‐day hESC‐developmental protocol (DPP) shown in Figure 1. In this protocol cells are induced, by serial growth factor applications, through a series of developmental steps to a primitive streak‐like stage, then to mesodermal progenitor followed by chondroprogenitors.^3^, ^4^, ^5^, ^18^

SIRT1 gene and protein expression significantly increased between days 2–5 of differentiation, then decreased steadily back to the original level by day 14 (Figure 1B,C). Alternate Sirtuins were also assessed; however, their transcript expression did not significantly change during the differentiation process (Figure 1B). In agreement with our previous studies,^5^ transcript expression for several chondrogenic genes increased steadily throughout the protocol (Figure 1D) including the core ECM components *COL2A1* and *ACAN*, *ARID5B*, and the major chondrogenic transcription factors *SOX5*, *SOX6* and *SOX9*. In parallel, we also observed an increase in SOX9 protein levels throughout the protocol (Figure 1C), while the hypertrophic marker *COLX* was not detected.

Since the histone acetyltransferase P300 and histone methyltransferase, SET Domain Containing 7 (SETD7) are associated with epigenetic regulation in chondrogenesis,^11^, ^28^ and both directly interact with SIRT1 on the *COL2A1* promoter,^29^, ^30^ these were also evaluated. Indeed, SETD7 may directly affect SIRT1 activity.^28^ A significant increase in SETD7 by day 14 of the protocol was observed, while no change in *P300* gene expression was seen (Figure 1E,F). Moreover, we observed no significant change in the gene expression of other chondrogenesis‐enhancing (Histone deacetylase 2 [*HDAC2*], 4 [*HDAC4*], histone acetyltransferase [*TIP60*, aka *KAT5*] and lysine demethylase 4B [*KDM4B*]), or repressing (DNA methyltransferase 1 [*DMNT1*] and lysine demethylase 2B, [*KDM2B*]) epigenetic modifiers (Figure S1A). While transcript and protein for the ARID5B cofactor, histone demethylase, PHF2,^22^, ^31^ were also detectable at day 14 of differentiation (Figure S1A,B). These data confirm that the increase in chondrogenic genes during differentiation is paralleled by changes in a subset of associated epigenetic effectors.

### 3.2 SIRT1 inhibition correlates with elevated *SOX9* transcription in hESC chondrogenesis

Next, we assessed the influence and timing of early SIRT1 activity on eventual chondrogenic differentiation by modulating SIRT1 activity during days 2–5 (stage1), or late from day 9 onwards (stage 3) of the monolayer (2D) protocol. Following the administration of the selective SIRT1 inhibitor EX527^13^, ^26^ during stage 1, *SOX9* gene expression increased slightly at day 14 compared to the same day control, however SOX9 protein level remained unchanged (Figure S1C,D). Inhibition of SIRT1 during stage 3 (i.e. from day 9–14) had no effect on expression of evaluated target genes at day 14 (Figure S1E). Activation of SIRT1 by the highly specific activator SRT1720^32^, ^33^ at either days 2–5 or from day 9–14 in all groups, did not result in any significant difference to control (Figure S1C,E). Together, the results of SIRT1 modulation during the early chondrogenic 2D differentiation protocol, support the idea that transition from mesoderm to a chondroprogenitor, during stages 1 and 3 of the protocol, does not require SIRT1 activity.

### 3.3 Activation of SIRT1 in a three‐dimensional (3D) pellet culture increased chondrogenic gene expression

As previous reports showed that SIRT1 modulation in a 3D setting resulted in augmented COL2 expression,^28^ we next took the D14 hESC‐chondroprogenitors and assessed them in a 3D culture system. Specifically, after 14 days of differentiation (Figure 1A) hESC‐chondroprogenitors were centrifuged, then cultured for 3 days to establish 3D pellets, after which the pellets were incubated in chondrogenic culture medium containing SIRT1 activity modulators SRT1720 or EX527 (Figure 2A). Results indicated that SIRT1 activation using 5 µM SRT1720, increased chondrogenic gene expression compared to untreated controls during the 7 days of pellet culture (Figure S2A).

Gene expression of pellets treated between 3 and 14 days after pelleting revealed a significant increase in expression of chondrogenic genes (i.e. *SOX5*, *ARID5B*, *COL2A1* and *ACAN*) in SRT1720 treated pellets compared to the starting chondroprogenitors (day 14 monolayer cultures), and untreated pellet controls. Conversely, there was a significant decrease in expression of the fibroblast/hypertrophy‐associated genes *COL1* and *RUNX2* in these pellets compared to untreated pellet controls (Figure 2B), while *COLX* was not detected in any of these conditions (*N* = 3, data not shown). Notably, these changes were not evident when pellets were treated with the activator for a shorter period of 3 days, from days 11 to 14 post pelleting (Figure S2B).

Gene expression for SIRT1 appeared visually to be increased after SRT1720 activation, however this was not significant (Figure 2B), while an independent epigenetic regulator, *DMNT1*, which is not associated with SIRT1, was unaffected (Figure 2B). Interestingly, gene expression for both *P300* and *PHF2* associated with SIRT1 action was significantly decreased after SRT1720 treatment compared to that in pre‐pelleted cells or untreated pellet controls (Figure S2). In contrast, the expression of *SETD7*, a factor which we have previously shown interacts with SIRT1,^28^ was significantly increased after SIRT1 activation (Figure S2C). However inhibiting SETD7 methyl‐transferase activity with (r)‐PFI‐2 in pellet culture did not affect chondrogenic gene expression compared to control (Figure S2D). Nor did it reduce the effect of SIRT1 activation by SRT (Figure 2E), indicating that SETD7 activity is likely not essential to SIRT1 action at this stage. Together, this data indicates that SIRT1 activation regulates recognized chondrogenic genes and may directly or indirectly regulate other epigenetic modulators during this process.

### 3.4 SIRT1 activation alters cartilage ECM expression and pellet histology

**FIGURE 3:**
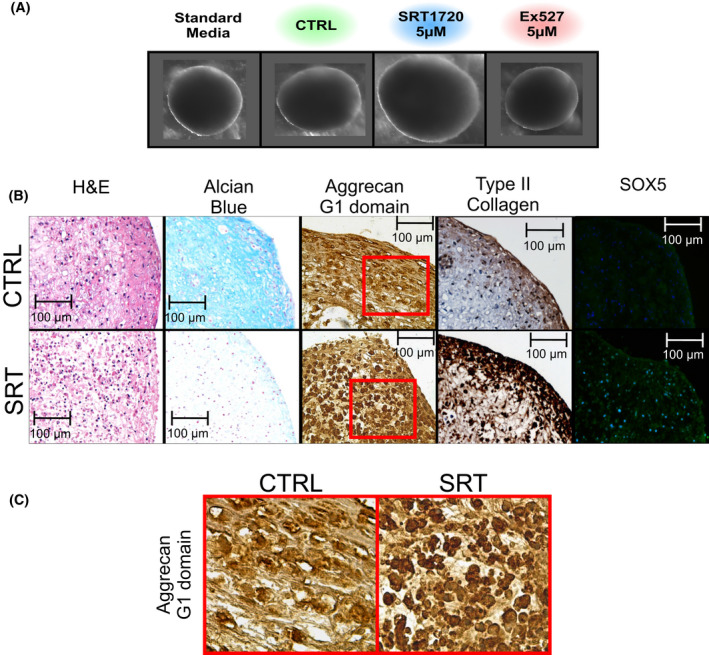
SIRT1 activation changes pellet structure and ECM component aggrecan expression. (A) Images of 3D pellet cultures at day 14 + 14 treated with vehicle controls or SIRT1 activity modulators; SRT1720 SIRT1 activator, or EX527 SIRT1 inhibitor from day 14 + 3 to 14 + 14. (B) IHC and pellet histology for structure (H&E) and GAG staining (alcian blue), immunohistochemical staining for aggrecan G1 domain, type‐II collagen, and immunofluorescence for SOX5 in day 14 + 28 pellets in 3D cultures treated ± SRT1720 (5 µM). (C) Enlarged view of aggrecan G1 domain staining. Position indicated by red outline

Prolonged SIRT1 activation (i.e. between 3 and 14 days after pelleting) resulted in enlarged pellets compared to controls, while pellets treated with EX527 did not display any change in size (Figure 3A). Histological analysis of control pellets displayed lacunae type structures, which are typically found in the developing joint cartilage, and which were abundant throughout the pellet, while elongated cells were observed towards the pellet surface, reminiscent of an articular surface (Figure 3B). Activation of SIRT1 reduced the abundance of these lacunae in the pellets and led to decreased alcian blue staining compared to control pellets (Figure 3B). However, there was a clear increase in staining for intracellular aggrecan with two different antibodies (Figure 3B,C, and Figure S3A). In addition to aggrecan, there was an increase in type‐II collagen (Figure 3, quantification in Figure S3B), SOX5 (correlating with its elevated gene expression in Figure 2B), and lubricin expression in SRT treated pellets (Figure S3A).

**FIGURE 4:**
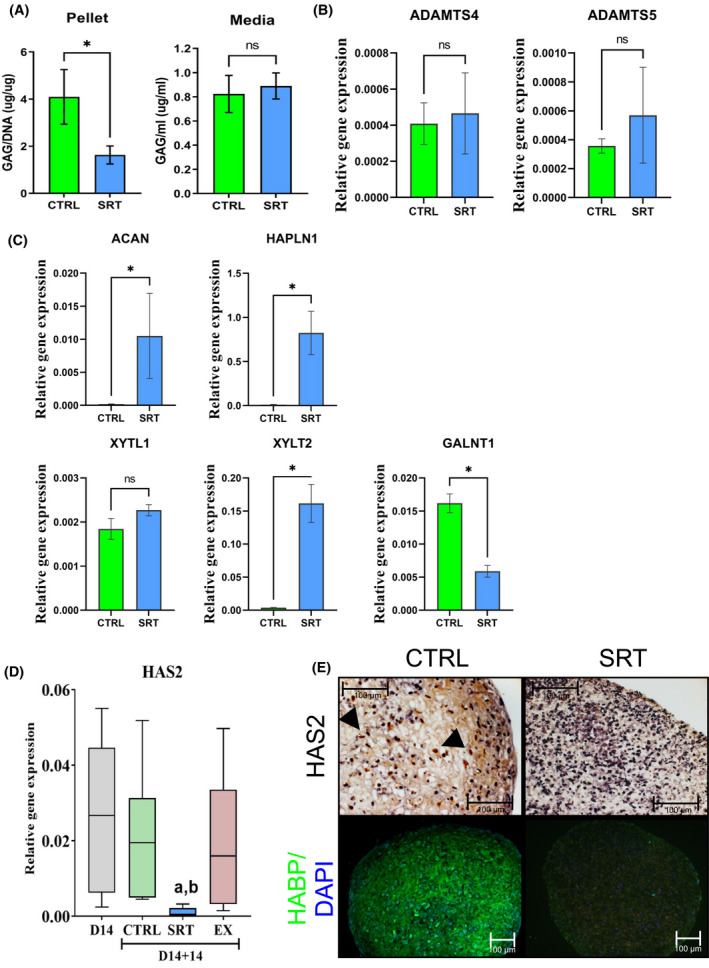
Activation of SIRT1 alters aggrecan synthesis and incorporation into the ECM. (A) DMMB GAG assay for D14 + 28 pellets and medium, after treatment with DMSO or SIRT1 activator SRT1720 (*N* = 3 biological repeats). * indicates significant difference to day 14 + 14 DMSO vehicle control (*p* ≤ .05) by ratio paired t‐test. (B) QRT‐PCR gene expression analysis of aggrecan degrading enzymes *ADAMTS4* and *ADAMTS5* in pellets at day 14 + 14, treated with DMSO vehicle or SIRT1 activator SRT1720 from day 14 + 3 to 14 + 14 (*N* = 3 biological repeats). (C) QRT‐PCR gene expression analysis of *ACAN* and synthesis associated factors (*HAPLN1*, *XYLT1* and *2*, and *GALNT1*) in pellets at day 14 + 14, treated with DMSO vehicle or SIRT1 activity activator SRT1720 from day 14 + 3 to 14 + 14 (*N* = 6 biological repeats). (D) QRT‐PCR gene expression analysis of *HAS2* in day 14 2D pre‐pellet cells, and pellets at day 14 + 14, treated with DMSO vehicle or SIRT1 activity modulators from day 14 + 3 to 14 + 14 (*N* = 7 biological repeats). Data displayed as gene expression relative to housekeeping gene *GAPDH* and shown as box plots. *a* indicates a significant difference to the Day 14 pre‐pellet sample (*p* ≤ .05), *b* indicates a significant difference to day 14 + 14 DMSO vehicle control (*p* ≤ .05). (E) IHC and IF pellet staining for type‐II collagen, HAS2 (example positive cells highlighted with arrows) in day 14 + 28 pellets, and HABP in day 14 + 28 pellets in 3D cultures treated ± SRT1720 (5 µM)

To understand the discrepancy between aggrecan protein presence and GAG content, the degradation and synthesis of aggrecan were assessed. In line with the alcian blue staining, pellet GAG content as measured by the DMMB assay was significantly decreased compared to DMSO vehicle control (Figure 4A). However, the GAG content in the medium was low and not significantly different between SRT1720 treated and control, suggesting there was no change in GAG release. Furthermore, there was no significant change in aggrecan degradation enzymes *ADAMTS4* or *5* to explain the decrease in GAG content within the pellet (Figure 4B).

Alongside the significant increase in *ACAN*, link protein (*HAPLN1*) gene expression was also increased after treatment with SRT1720, indicating aggrecan had the means to bind to hyaluronic acid (HA) (Figure 4C). There was also a significant increase in gene expression of xylosyl‐transferase isoform 2 (*XYLT2*), responsible for the addition of a binding site for GAG groups on aggrecan^34^ (Figure 4C). In contrast, there was a significant decrease in expression of the polypeptide GalNAc transferase 1 gene, *GALNT1*, which codes for an enzyme that initiates formation of GAG chains in the modification and maturation of aggrecan in the Golgi,^34^ and which is highly expressed in developing cartilage.^35^ Similarly, the major enzyme driving synthesis of HA, HAS2, was significantly reduced after SIRT1 activation (Figure 4D,E) resulting in a reduction in HA itself (Figure 4E). Together this data indicates a differential regulation by activated SIRT1 between chondrogenic proteins and GAGs during hESC‐chondrocyte differentiation.

### 3.5 Activation of SIRT1 in TC28a2 3D pellets enhances chondrogenic gene expression

**FIGURE 5:**
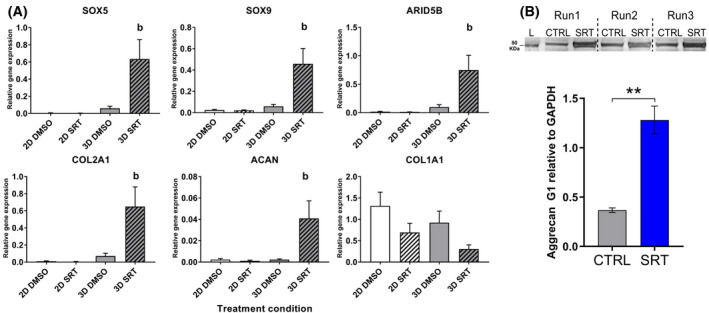
Activation of SIRT1 in TC28a2 cells enhances chondrogenic gene expression in 3D but not 2D culture. (A) QRT‐PCR gene expression analysis of chondrogenesis associated genes in TC28a2 immortalized chondrocytes in 2D or 3D culture for 7 days, treated with DMSO vehicle (control) or SIRT1 activator SRT1720 (5 µM) from days 3 to 7 (*N* = 3 biological repeats). (B) Western blot protein expression analysis for the G1 domain of aggrecan in TC28a2 14‐day old pellets treated with DMSO or SRT1720 from day 3 till 14, and quantification of densitometry (*N* = 3 biological repeats). Gene expression data displayed as relative to housekeeping gene *GAPDH*. *b* indicates a significant difference to 3D DMSO vehicle (control) (*p* ≤ .05). ** indicates a significant difference (*p* ≤ .01) compared to control

Given that SIRT1 activation of hESC‐chondroprogenitors induced chondrogenic gene expression in 3D pellets, we investigated whether this response was dependent on developmental maturity using a mature chondrogenic cell line (TC28a2). Monolayer or pelleted TC28a2 cells were cultured for 3 days, before treatment with SRT1720 or DMSO vehicle control for an additional 4 days. Results indicated a small but insignificant increase in chondrogenic gene expression in TC28a2 pellets compared to monolayer cultures (Figure 5A). SIRT1 activation in TC28a2 pellets induced a significant increase in transcript for chondrogenic genes compared to the control (Figure 5A) and led to larger pellets (data not shown), while there was no observable change in gene expression in SRT1720 treated monolayer cells. *COL1A2* expression was not affected by SIRT1720 in either culture format. Assessment of aggrecan protein levels by western blotting indicated a substantial increase in the band at 80KDa,^36^ in line with the increased level of *ACAN* gene expression (Figure 5B). Hence, these results show that SIRT1 activation increased *ACAN* gene expression and protein during chondrogenesis in a 3D setting, like in hESC‐chondroprogenitor pellets. Indeed, using SIRT1 shRNA led to a reduction in *ACAN*, *SOX9*, *SOX5* and *ARID5B* with a trend to decrease in *COL2A1* after SIRT1 knock down in SIRT1 activated chondrogenic cells (Figure S3D,E).

### 3.6 SIRT1 activity, not expression level, drives chondrogenic gene expression

**FIGURE 6:**
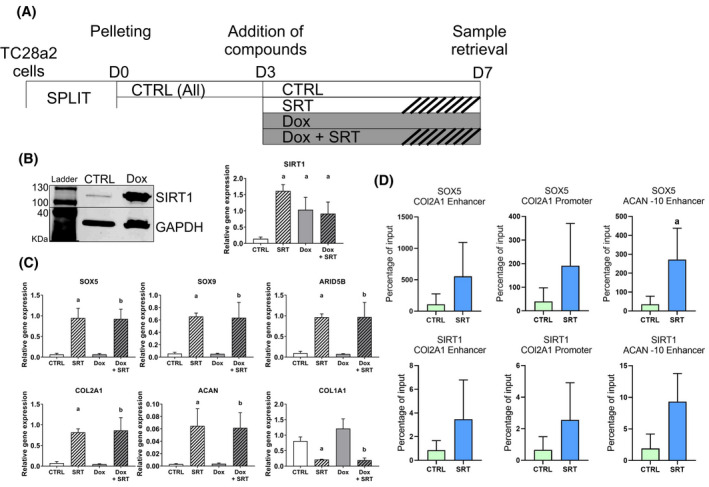
SIRT1 activation is important for promotion of chondrogenic genes. (A) Schematic diagram of TC28a2 pellet culture and conditions. Bar designs for each condition are conserved between panels A, B and C. (B) Western blot analysis of SIRT1 protein expression in Dox responsive SIRT1 overexpressing TC28a2 cells, treated with or without 100 µg/ml Dox from day 3 till day 14. (C) QRT‐PCR gene expression analysis of chondrogenic genes in Dox responsive SIRT1 overexpressing TC28a2 pellets cultured for 14 days (*N* = 4 biological repeats). Pellets were treated from day 3 till day 14 with DMSO (control), SRT1720 (5 µg), Dox (100 µg/ml) or a combination of Dox and SRT1720. Data displayed relative to housekeeping gene *GAPDH*. (D) ChIP analysis of SIRT1 and SOX5 occupancy of the *COL2A1* enhancer and promoter region, and of the *ACAN‐*10 enhancer region in day 14 + 14 pellets derived from hPSC‐chondroprogenitors (*N* = 3 biological repeats). Data displayed as percentage of input. *a* indicates a significant difference (*p* ≤ .05) to DMSO (control). *b* indicates a significant difference (*p* ≤ .05) to Dox treated control

Based on the above results, we assessed whether overexpression of SIRT1 would increase chondrogenic ECM expression, as shown above for SIRT1 chemical activation. To this end we transfected TC28a2 cells with a Dox‐inducible SIRT1 overexpression construct and cultured the cells in 3D pellets with or without SRT1720 (Figure 6A). Stimulation of the TC28a2 pellets with 100 nM Dox was sufficient to elicit a large increase in SIRT1 protein levels (Figure 6B). However, SIRT1 overexpression without SIRT1 activation did not increase chondrogenic gene expression (Figure 6C). Simultaneous SIRT1 overexpression and activation generated increases in transcripts for the chondrogenic genes, almost identical to those seen with SRT1720 activation alone (Figures 2 and 6C). This suggests that SIRT1 protein abundance is not the rate limiting factor in inducing chondrogenic gene expression. Rather, additional factors, acting in concert with activated SIRT1, contribute to its function and enhancement of a chondrogenic phenotype in a 3D setting.

### 3.7 SIRT1 activation leads to SOX5 enrichment at the *ACAN‐*10 enhancer site

As SOX5 was significantly increased during SIRT1 activation in pellet culture, we further assessed the dynamics between SIRT1 and SOX5 in regulating *ACAN* expression. To this end, we carried out ChIP analysis in TC28a2 cells for SIRT1 and SOX5 in day 14 + 14 chondroprogenitor pellets with or without stimulation with SRT1720, focusing on the ‐10 enhancer site for *ACAN*. This chondrocyte specific *ACAN* enhancer is located 10kb upstream of the *ACAN* transcription start site as a highly conserved region also known as the A1 site.^20^ PCR analysis of the ChIPed DNA revealed significant enrichment of SOX5 at the *ACAN* ‐10 enhancer region, with SIRT1 showing a trend towards enrichment (*p* = .06) following SRT1720 activation (Figure 6D). The data suggest that SIRT1 contributes to *ACAN* expression by enrichment of SOX5 on the enhancer site of the gene.

### 3.8 ARID5B is required for type‐II collagen gene expression in SIRT1 activated chondrogenic pellets

**FIGURE 7:**
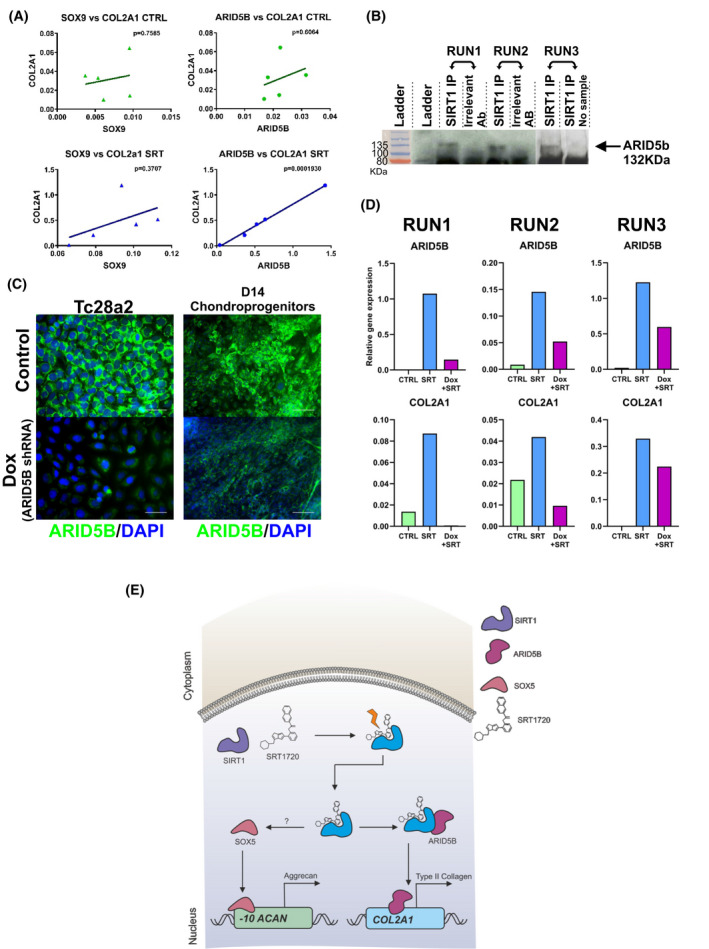
ARID5B is required for *COL2A1* expression in SIRT1 activated pellets. (A) Regression curve and Pearson's r correlation p value analysis of chondrogenic transcription factors, *SOX9* or *ARID5B* vs *COL2A1* gene expression (*N* = 5 biological repeats) in day 14 + 14 pellets culture with DMSO (control) or SRT1720 from day 14 + 3 to 14 + 14. (B) Western blot analysis of ARID5B in SIRT1 or irrelevant antibody (P300) immunoprecipitate from day 14 + 14 pellets treated with either DMSO (control) or SRT1720 from day 14 + 3 till day 14 + 14. (C) Immunocytochemistry of ARID5B in Dox responsive *ARID5B*‐shRNA TC28a2 cells and day 14 hPSC derived chondroprogenitors, treated with DMSO (control) or 100ng/ml Dox for 48 hours. (D) Gene expression analysis of *ARID5B* and *COL2A1* in Dox responsive *ARID5B*‐shRNA hPSC derived chondroprogenitor pellets cultured for 14 days in 3D, treated with DMSO (control), SRT1720 (5 µM), or SRT1720 (5 µM) and Dox (100 ng/ml) from day 14 + 3 till 14 + 14. Gene expression data displayed relative to housekeeping gene *GAPDH*. (E) Models for SIRT1 activation, including impact on downstream associated factors and resulting ECM component expression

Importantly, the gene expression of *ARID5B* was significantly increased in SIRT1 activated pellets (Figure 2B) alongside *SOX5*. Thus, we investigated whether it was likely to be involved in the increased levels of aggrecan and type‐II collagen observed in the developing 3D hESC‐cartilage pellet culture. Pearson's correlation analysis between *COL2A1* and *SOX9*, or *ARID5B* exhibited a weak correlation in control 3D culture. However, after SIRT1 activation we observed a significant correlation (*r* ^2^ = 0.994, *p* = .0002) between *COL2A1 and ARID5B* (Figure 7A).

To assess any direct binding between ARID5B and SIRT1 we employed co‐IP, with P300 used as an irrelevant antibody control. Western blot analysis of co‐IP samples probed with the ARID5B antibody, displayed a band at approximately 135KDa (ARID5B reported size; 132Kda), detectable only in the SIRT1 immunoprecipitated lanes (Figure 7B). This band was not present in the co‐IP control lanes nor in the SIRT1 IP only control (Supplementary Figure 3F).

To support this correlation of SIRT1 activation with ARID5B function, a Dox inducible *ARID5B*‐shRNA construct was utilized. Upon Doxycycline stimulation ARID5B protein levels were suppressed in both TC28a2 cells and day‐14 hESC‐chondroprogenitors (Figure 7C). When stimulated with SRT1720 and Doxycycline, the resultant decrease in ARID5B, was reflected in a subsequent reduction in *COL2A1* expression (Figure 7D) not seen in controls. These data indicate that SIRT1 stimulates COL2A1 expression possibly in‐part via a novel mechanism involving ARID5B.

## 4 DISCUSSION

The deacetylase enzyme SIRT1 has been implicated as an important regulator of cartilage homeostasis.^11^, ^13^, ^15^ This study aimed at understanding the involvement of this enzyme and the timing of its activity during human chondrocyte development by using hESCs.

SIRT1 is important in the maintenance of pluripotency, interacting with multiple pluripotency associated pathways.^37^, ^38^, ^39^, ^40^, ^41^ Indeed, SIRT1 inhibition in hPSCs reduced stemness and promoted neuronal differentiation.^42^, ^43^, ^44^ As such, the notable SIRT1 expression observed in hESCs was expected. Furthermore, an increase in SIRT1 during days 2–5 of the protocol, may reflect mesodermal lineage commitment.^45^ However, this may not be deacetylase activity‐related, as activation or inhibition of SIRT1 during this period did not promote (or impair) hESC mesodermal differentiation in our study.

This study has generated new insight into human chondrogenic differentiation from hPSCs; especially by extending into a 3D pellet format. Though pellet culture of hPSC derived chondroprogenitors has been achieved previously,^46^, ^47^, ^48^ our study utilized a defined development system, without selection from embryoid bodies^48^ or transition through an MSC‐like intermediate, nor the associated hypertrophic/fibroblastic gene expression.^49^ Importantly, our results show pellet culture cells have enhanced chondrogenic gene expression after 14 days, with a cartilage‐like, alcian blue positive ECM emerging after 28 days in pellet.

In striking contrast to 2D, activation of SIRT1 during the 3D pellet culture significantly increased the main chondrogenic proteins in the chondroprogenitors, while decreasing the fibrotic and hypertrophic factors. This finding was also replicated during activation of SIRT1 in TC28a2 cultured in 3D. Altered SIRT1 activity and responsiveness between 2D and 3D culture has been shown in primary human chondrocytes,^28^ and it was proposed that the differential response stemmed from cell de‐differentiation in 2D or redifferentiation in 3D respectively. Though no study has demonstrated this in development, we showed previously that the transition of hESC‐chondroprogenitors from 2D to 3D aggregates, correlates with higher COL2A1 expression.^5^ Thus, the change in responsiveness to SRT1720 in 3D indicates that 3D cell architecture and/or cell contact is an essential factor for its effect on chondrogenic expression.

While activation of SIRT1 dramatically increased several chondrogenic transcription factors and ECM components, SIRT1‐inhibited samples were able to differentiate into chondrocyte‐like cells and produce ECM proteins much like non treated controls. Crucially, this is in line with *Sirt1* ^−/−^ mouse KO models,^17^ which do form cartilage but have an altered cartilage phenotype and express decreased levels of type‐II collagen and aggrecan. This supports the idea that SIRT1 deacetylase activity is not essential for chondrogenic gene expression per se, but instead is an important epigenetic regulator facilitating the synthesis of adequate matrix protein during human cartilage development. This is apparent in the elevated type‐II collagen and aggrecan levels (with associated increase in *HAPLN1* coding link protein) in our SIRT1 activated samples. However, in parallel we observed a decrease in GAG measured in the matrix, which was reflected in the down regulation of *HAS2* (responsible for HA synthesis) and *GALNT1*; responsible for chondroitin sulphate GAG chain initiation.^34^ Any abnormality in GAG assembly may be further compounded by an unbalanced substantial upregulation of *XYLT2*, which is involved in formation of the initiating GAG chain tetrasaccharide, and has been shown to influence glycosylation site pattern.^50^ This would suggest the retention of core aggrecan protein in the cells and ECM GAG content is likely the result of a reduction in the secretion of mature GAGylated aggrecan by the SRT1720 treated cells, alongside a lack of HA available for any mature aggrecan to bind to. Whether this reflects direct regulation though SIRT1 activation or an indirect feedback mechanism cannot be determined.

Here we uncovered that SIRT1 overexpression combined with activation did not increase chondrogenic gene expression beyond activation alone, suggesting adequate amounts of SIRT1 in the cells but a deficit of activation factor(s). Results show that SRT1720 activation alone led to significant increases in SIRT1 expression, and that this was not increased any further by the overexpression system. This could indicate feedback of SIRT1 activity on SIRT1 expression, which overrides the influence of SIRT1 overexpression in this system. So SIRT1 activation may positively affect mediating factors and these may be the limiting factors in SIRT1’s influence on chondrogenic protein expression. Activation with SRT1720 is therefore not limited by the availability of SIRT1 in this system but the factors with which it associates.

In this context SIRT1 activation led to significant increases in the expression of SOX9 associated co‐factors, in particular SOX5^51^ and ARID5B,^22^ indicating their potential importance as downstream regulators of chondrogenesis. There was a significant enrichment of SOX5 (with a trend to SIRT1 enrichment) at the ACAN‐10 enhancer, in line with previous observations for SOX9.^13^ This together with the demonstration of SIRT1‐ARID5B association, suggests that SIRT1 is reliant on these downstream factors to regulate gene transcription as summarized in Figure 6E.

SOX5 binds to and co‐directs chondrogenesis in combination with SOX9,^51^, ^52^ as part of the SOX trio required for permanent cartilage.^19^ SIRT1 has also been shown to deacetylate multiple SOX family members,^37^, ^38^ including SOX9, facilitating its translocation into the nucleus, and thereby promoting chondrogenic activity.^13^ During chondrogenesis SIRT1 is able to bind both COL2A1 and ACAN promoter/enhancer sites,^28^ as does SOX5, specifically at the ‐10 enhancer region.^20^ Indeed Lefebvre and Dvir‐Ginzberg surmised that there are several conserved lysine residues between the SOX family members^21^ which would be potential targets for SIRT1 modification, indicating that SIRT1 is likely to regulate nuclear entry of SOX family proteins to facilitate their transcriptional activity.

Here we report, that ARID5B is also expressed in hESC‐chondrogenic pellets undergoing differentiation. ARID5B is reported to form a complex with PHF2, directing factors such as SOX9.^22^ In this study ARID5B expression also correlated significantly with chondrogenic ECM proteins, and these observations together with the inferred regulatory roles suggest further potential drug targets to manipulate human chondrogenesis and promote healthy skeletal development.

Our protocol generates moderate amounts of type‐II collagen.^4^, ^5^ Recent papers have also generated chondrocytes with good type‐II collagen from hPSC using protocols aimed at articular‐like cartilage,^48^, ^53^ and others from iPSC‐iMSC growth plate like regimens.^54^, ^55^ Importantly, SIRT1 activity has been widely reported as beneficial to bone marrow MSC‐chondrogenesis.^12^ Indeed, SIRT1 activity may have contributed to the synthesis of type‐II collagen seen in those protocols, however this was not assessed. In this current study we show dramatic changes in chondrogenic genes (e.g. *SOX5*, *SOX9*, *ARID5B*, *ACAN*, and *COL2A1*) through the activation of SIRT1 which are also reflected in type‐II collagen and aggrecan protein, but decreased GAGS paralleled by changes in gene expression for glycosylation enzymes. Results of this study indicate that epigenetic factors such as SIRT1 are of great relevance to other protocols for the musculoskeletal differentiation of hPSCs. Indeed, this information will be utilized to improve our current protocol.

While the current study demonstrates the role of SIRT1 in chondrogenesis during a human developmental system, it poses additional questions which we hope to fully answer in the future. Importantly, there are several factors which can affect SIRT1 activity, which were not assessed in this study including inhibitory factors such as AROS,^56^ post translational modifications,^57^ as well as cleavage of SIRT1^58^; which may reduce its activity levels, even under overexpression conditions. Assessing the activity of SIRT1, either directly or by relative NAD (substrate) availability, during hESC‐chondrogenesis in future studies may help to understand the influence of these factors. Additionally, the importance of ARID5B in chondrogenesis is only just beginning to be understood. Therefore its relationship to SIRT1 is intriguing and warrants further research in greater detail than presented in the current study.

In summary this work has identified a critical role for SIRT1 in control of ECM protein expression in hESC‐chondrocyte development. It has also identified and refined our understanding of the role of several additional factors regulating human chondrogenic development. We have shown that, in this human model of chondrogenic development, SIRT1 activation differentially increases gene expression for ECM proteins including the core components type‐II collagen and aggrecan, while reducing GAG formation. This regulatory effect is stage dependent and requires a 3D arrangement of developing chondroprogenitors. Additionally, we have shown that after SIRT1 activation, *ARID5b* and *COL2a1* both increase in developing chondroprogenitors and changes in these genes are directly correlated, suggesting co‐regulation. Finally, SIRT1 induction of *COL2A1* expression is ARID5B‐dependent, and we identify a potential novel mechanism for control of cartilage protein synthesis involving binding of ARID5B to SIRT1. Thus, SIRT1 activity can benefit cartilage development but only if the positive effects on chondrogenic proteins can be balanced by other positive modulators of GAG synthesis.

## CONFLICT OF INTEREST

The authors state that there is no conflict interest in connection with this article.

## AUTHOR CONTRIBUTIONS

Study design and concept by Mona Dvir‐Ginzberg and Susan J. Kimber. Experimental design and undertaking by Christopher A. Smith. Cell culture by Christopher A. Smith and Nicola Bates. Additional samples supplied by Paul A. Humphreys and Mark A. Naven. Viral construct design and production by Stuart A. Cain. Data analysis by Christopher A. Smith, Susan J. Kimber and Mona Dvir‐Ginzberg. Manuscript written and edited by Christopher A. Smith, Susan J. Kimber and Mona Dvir‐Ginzberg.

## Supporting information

Fig S1‐S3

Table S1‐S2

## Data Availability

The data that support the findings of this study are available from the corresponding authors upon reasonable request.
