# Supplementary material for: SIRT1 activity orchestrates ECM expression during hESC‐chondrogenic differentiation
Source: FASEB J. 2022 Apr 13;36(5):e22314. doi: 10.1096/fj.202200169R (PMC9322318; doi:10.1096/fj.202200169R)
Supplement: Supplementary file 1 — Fig S1‐S3 [file FSB2-36-0-s001.docx]

**
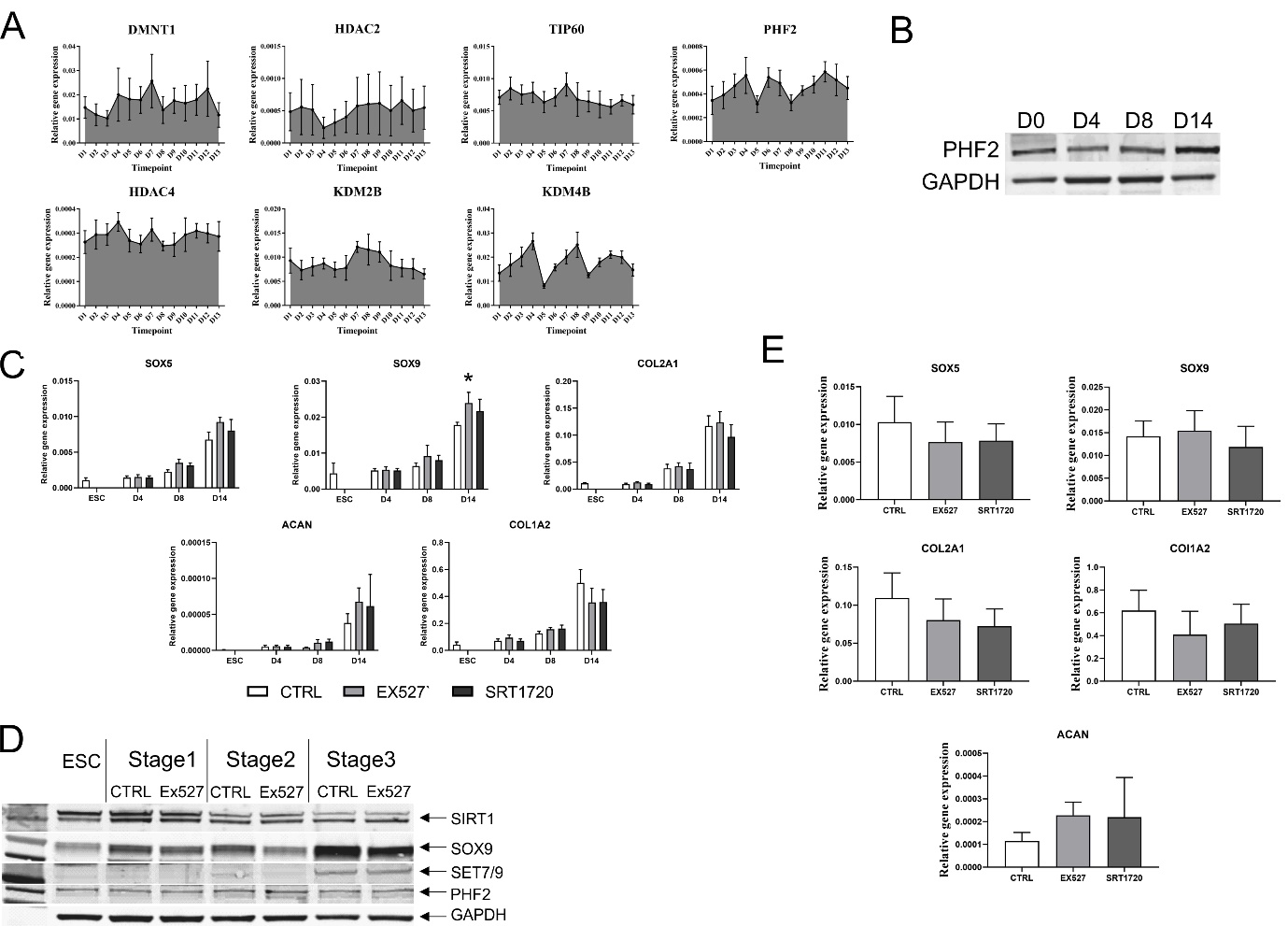
**

**Figure S1: Influence of SIRT1 modulators on early hESC mesoderm and chondroprogenitor differentiation during 2D culture.** A) QRT-PCR gene transcription analysis of alternate epigenetic factors associated with initial hESC-chondrogenesis in the 14-day DDP (N=4 biological repeats). B) Western blot protein expression analysis of PHF2 and GAPDH for cell samples taken at days 0, 4, 8 and 14 during the chondroprogenitor differentiation protocol. C) QRT-PCR analysis of samples taken for hESCs, end of stage 1 (day 4), end of stage 2 (day 8) and end of stage 3 (day 14) of the 2D chondroprogenitor differentiation protocol for cells treated with the selective SIRT1 activator, SRT1720, or inhibitor, EX527 between days 2-5 of the chondrogenic protocol (N=4 biological repeats), and D) Western blot protein expression analysis of SIRT1, SOX9, SETD7, PHF2 and GAPDH for cell samples taken at day 0, 4, 8 and 14 for cells treated with 5 µM EX527 during days 2-5 of the protocol (N=1). E) QRT-PCR analysis of samples taken at the end of stage 3 (day 14) of the 2D chondroprogenitor differentiation protocol for control cells or cells treated with selective SIRT1 activator SRT1720 or inhibitor EX527 from day 8 onwards (N=4 biological repeats). All gene expression data displayed relative to housekeeping gene *GAPDH.* * signifies significant difference (p≤0.05) compared to same day control.

**
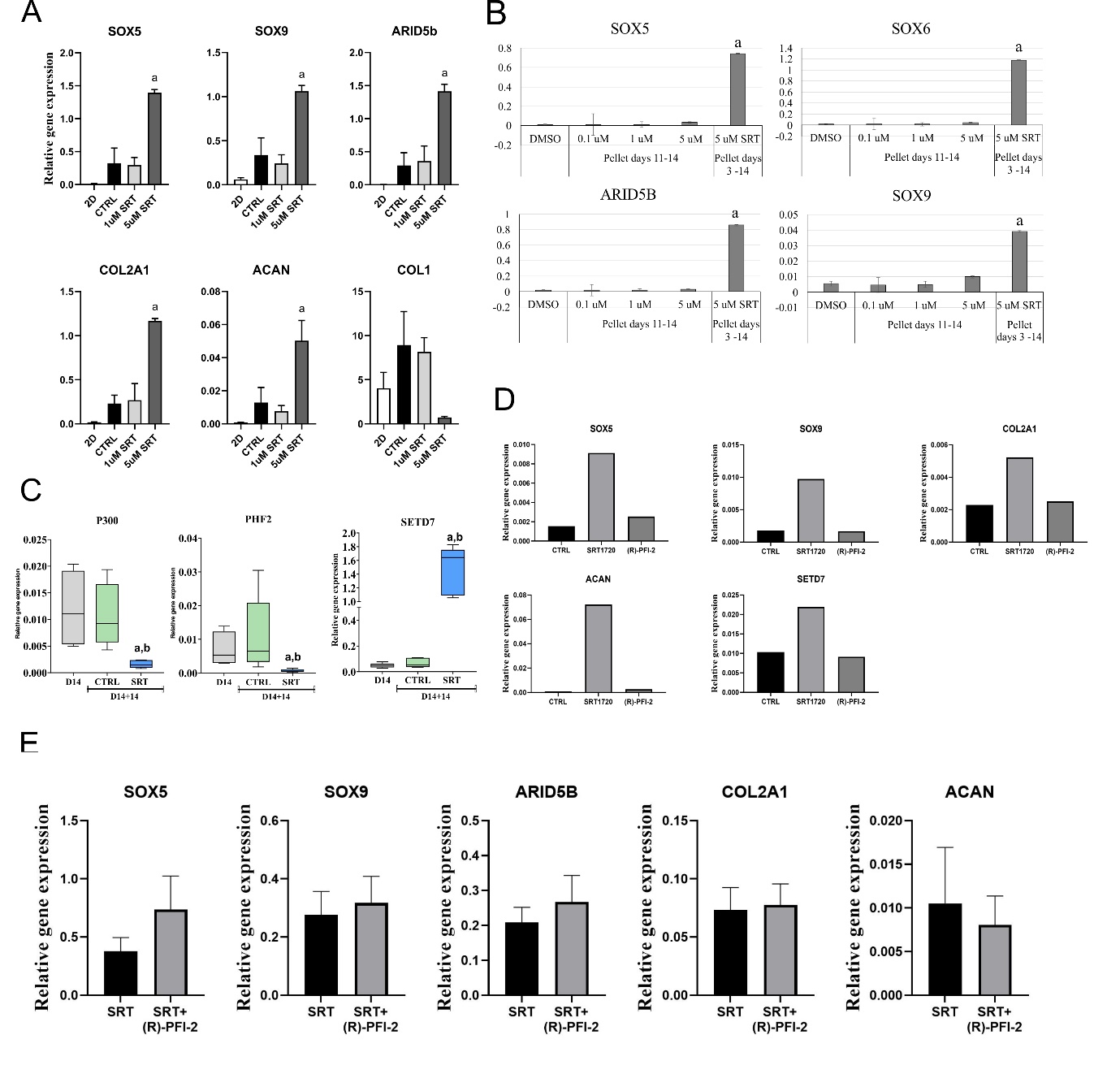
 Figure S2: Effect of epigenetic modulators in 3D pellet culture.** A) Gene expression analysis of day 14+7 chondrogenic pellets treated with 1 or 5 µM SRT1720 for 4 days from day 14+3 (N=3 biological repeats). B). QRT-PCR gene expression analysis of chondrogenic transcription factors in pellets treated with DMSO vehicle, or SRT1720 from days 3-14 (11 days) or from days 11-14 (3 days) (N=5 biological repeats). SRT1720 concentrations as displayed. C) QRT-PCR gene expression analysis of epigenetic factors in 2D day 14 (pre-pellet) cells, and pellets at day 14+14, treated with DMSO vehicle or SIRT1 activator SRT1720 (5uM) from day 14+3 to 14+14 (N=7 biological repeats). D) QRT-PCR gene expression analysis of day 14+14 pellets treated with DMSO vehicle control (CTRL), SIRT1 activator SRT1720 (5 µM), or SETD7 inhibitor (r)-PFI-2 (1 µM) from days 14+3 till 14+14 (N=1). E) QRT-PCR gene expression analysis of chondrogenic factors in pellets at day 14+14, treated with SIRT1 activator SRT1720 (5uM) singularly or in combination with SETD7 inhibitor (R)-PFI-2 (1uM) from day 14+3 to 14+14 (N=3 biological repeats). Gene expression data displayed as relative to housekeeping gene *GAPDH. a* signifies significant difference (p≤0.05) compared to DMSO vehicle control (CTRL).

**
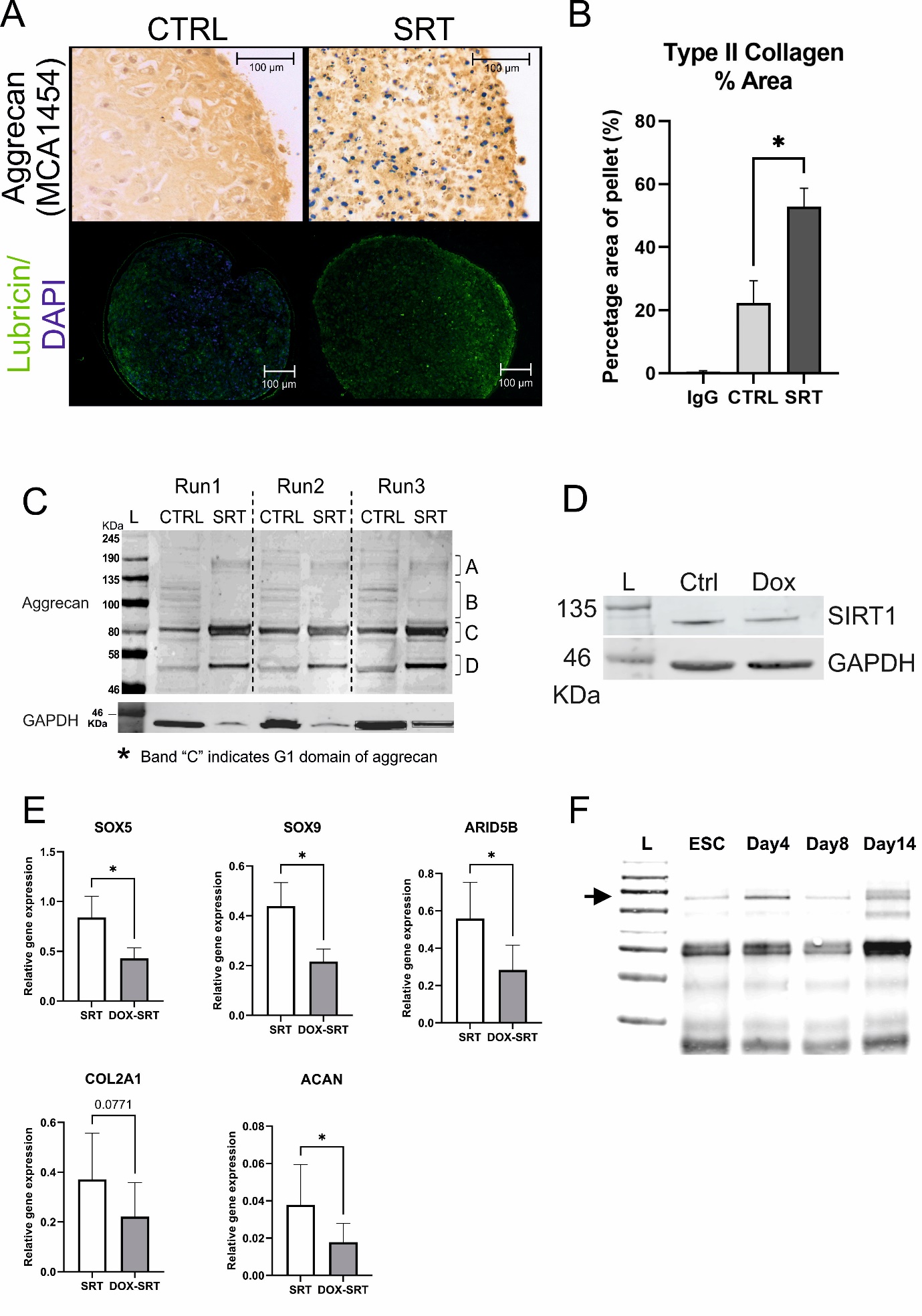
 Figure S3. Activation of SIRT1 changes balance in expression of ECM components.** A) IHC pellet staining for aggrecan with alternative antibody (MCA1454) and IF staining for lubricin in day 14+28 pellets in 3D cultures treated +/- SRT1720 (5 µM). B) Semi-quantification of type-II collagen staining in IHC sections. Displayed as area of DAB positive pellet staining after probe with IgG isotype, or type-II collagen antibody, for sections from day 14+28 pellets in control (CTRL, N=4 biological repeats) and SRT1720 treated (SRT, N=5 biological repeats). * Signifies significant difference (p≤0.05) between treatments. C) Western blot protein expression analysis for aggrecan, and GAPDH housekeeping gene in TC28a2 14-day old pellets treated with DMSO or SRT1720 (5 µM) from day 3 till 14 (N=3 biological repeats) (full blot of that shown in Figure 5). Bands correspond to sizes detailed in original paper [29], G1 domain indicated in band C. D) Western blot for SIRT1 (135KDa) and GAPDH (46KDa) housekeeping gene for cells transduced with doxycycline (DOX) responsive shRNA for SIRT1, treated with or without 100nM Dox. Blot quantification indicates a reduction of 30%. E) Gene expression analysis of chondrogenic factors in Dox responsive SIRT1-shRNA pellets of chondrogenic cells cultured for 14 days in 3D, treated with SRT1720 (5 µM), or SRT1720 (5 µM) and Dox (100 ng/ml) from day 14+3 till 14+14. Gene expression data displayed relative to housekeeping gene *GAPDH.* * indicates significant difference between conditions (p≤0.05) by ratio paired t-test. F) Western blot analysis for SIRT1 in SIRT1 IP samples taken from ESC and differentiating cells during the 14-day protocol, at day 0 (ESC), day 4, day 8 and day 14. Arrow indicates SIRT1 presence at approximately 110KDa.
